# Value of a confirmatory re‐biopsy as part of a modern risk stratified cancer surveillance programme for early prostate cancer

**DOI:** 10.1002/bco2.406

**Published:** 2024-06-01

**Authors:** Harry Gabb, Vincent J. Gnanapragasam

**Affiliations:** ^1^ Clinical School of Medicine University of Cambridge Cambridge UK; ^2^ Cambridge Urology Translational Research and Clinical Trials Office Cambridge UK; ^3^ Division of Urology, Department of Surgery University of Cambridge Cambridge UK

**Keywords:** active surveillance, Cambridge Prognostic Groups, confirmatory biopsy, prostate cancer, risk‐based follow‐up, stratified cancer surveillance

Active Surveillance (AS) is an important management strategy for patients diagnosed with early prostate cancer. Current National Institute for Health and Care Excellence (NICE) guidelines recommend AS as first‐line for patients in Cambridge Prognostic Group 1 (CPG1), as an equal option for CPG2 and an alternative for those with CPG3 who decline radical treatment (https://www.nice.org.uk/guidance/ng131). The elements of modern AS incorporate regular prostate‐specific antigen (PSA) tests and MRI scans at defined timepoints. Early repeat or confirmatory biopsies, however, are not currently mandated in any modern guidance or protocol. Previous research has shown that first diagnostic biopsies can under‐represent disease burden in men on AS and is improved if pre‐biopsy MRI guidance is used.^1^, ^2^ We have previously published that MRI pre‐biopsy improves disease characterisation at diagnosis that can translate into lower rates of AS progression compared with benchmark series.^3^ However, the value of confirmatory/early re‐biopsy in addition to pre‐MRI diagnostics remains unknown. This is particularly important if AS follow‐up is to be individualised at its commencement based on predicted disease behaviour, progression risk and to minimise future use of protocol re‐biopsies.^4^

We have developed and implemented the Stratified Cancer Active Surveillance (STRATCANS) programme in our centre details of which we have reported and are in this webtool https://stratcans.com.^4^, ^5^ In summary, STRATCANS defines three tiers of follow‐up based on the risk of progression determined by diagnostic CPG, PSA density and MRI. In STRATCANS, men with the least burden of disease (STRATCANS tier‐1, that is, CPG1 and a low PSA density) are managed primarily by PSA (which can be patient self‐monitored) and may not need clinical review for up to 18 months. MRI repeat is based on daignostic PIRADS score and biopsies used only if there is a change. This light touch review mandates that the disease burden at the start of AS is very well characterised. Given this context the goal of this study was to assess the value of confirmatory re‐biopsy in STRATCANS in confirming the disease burden at the start of AS and hence allocation to the correct STRATCANS tier.

A retrospective case notes review of men on STRATCANS who agreed to confirmatory re‐biopsy (within 12 months of diagnosis) was performed as part of an ongoing service evaluation of STRATCANS in our unit (Cambridge University Hospitals NHS Foundation Trust, Cambridge, UK; registration number: PRN11857). Men were routinely offered early re‐biopsy when selecting AS management unless unfit for another procedure. Data from all men who agreed are included here, and there were no other case selections applied. The only other exclusion was therefore if a patient declined based on their own preferences. Those with significant co‐morbidity and limited life expectancy were placed on watchful waiting and not included in STRATCANS. All men had initially had cognitive or fusion image‐guided diagnostic prostate biopsy with pre‐biopsy MRI, and the quality of our service has been well documented.^6^ Repeat biopsy were by a transperineal template approach under general anaesthetic (GATP) between August 2018 and June 2022. One was done under LA for co‐morbidity reasons. We recorded diagnosis data including PSA, prostate volume and PSA density, PIRADs score, percentage core positivity, route of first biopsy (LATP–transperineal or transrectal–TRUS), Grade Group (GG) and CPG assignment. Outcome measure included changes in GG and CPG following re‐biopsy (i.e., reclassification), cancer core positivity and any factors that may predict reclassification. Student's *t* tests and chi‐square analysis were performed using proprietary software.

Data from 40 men were analysed in the study. The demographic characteristics of the cohort are shown in Table 1. Median PSA and PSAd were 6.2 ng/mL and 0.14 ng/mL^2^, respectively, and 33/40 had PIRAD 4–5 lesions on MRI pre‐biopsy (82.5%). At diagnosis, 22/40 (55%) were GG1 and 18 were GG2 (45%). Twenty‐one were classed as CPG1 and 17 were CPG2, with 2 classed as CPG3 based on GG2 and PSA > 10. The median number of days between initial biopsy and re‐biopsy was 201. Overall, 15/40 (37.5%) men were upgraded (and CPG reclassified) following re‐biopsy, while 6/40 (15%) had a decrease in GG (Table 2). Of the men upgraded, 5/40 men (12.5%) were re‐graded to ≥GG3 with one case each re‐graded to GG4 or 5 (5%) and hence were no longer suitable for AS. 20/40 (50%) had higher biopsy percentage core positivity compared with initial diagnostic biopsies (Table 2). Of these 15 up‐classifed men, 7/15 opted to have treatment instead of continuing AS. The remaining eight men were moved to a more intense STRATCANS follow‐up tier. Higher median PSA, PIRADS and PSAd and use of transperineal biopsy method at diagnosis trended to be associated with upgrading however none reached statistical differences (Table S1A). Finally, we tested for any temporal differences in the rates of reclassification to account for any changes in diagnostic biopsy method or personnel over time which may have led to a change in our detection quality and skewed our results. To achieve this, the cohort was divided into two time periods, 2018–mid 2021 and 2021–2022, as well as by three time periods 2018–2020 (pre‐pandemic), 2021 and 2022. In this analysis, we found no difference in rates of upgrading, CPG reclassification or changes in percentage core positivity using two time periods (Table S1B) (*p* = 0.74 and 0.12, respectively). Similarly, we found no significant changes with further splitting the cohort into three time periods (*p* = 0.30 and 0.49, data not shown).

**TABLE 1:** Demographics of the population of men who underwent confirmatory or early re‐biopsy (<12 months from diagnosis) after entry into the Stratified Cancer Active Surveillance (STRATCANS) programme in our centre.

Variable	Median	Mean	Range
Age (years)	67.2	67	51–80
PSA (ng/mL)	6.2	6.4	1.3–12.9
PIRADS score	4	‐	1–5
	PIRADS 1–2 (4)		
	PIRADS 3 (3)		
	PIRADS 4 (18)		
	PIRADS 5 (15)		
Prostate volume (mL)	40	47.8	16–125
PSA density (ng/mL^2^)	0.14	0.16	0.04–0.26
Diagnostic Grade Group (GG)	1	‐	1–2
	GG1 (22)		
	GG2 (18)		
Diagnostic CPG	1	‐	1–2
	CPG1 (21)		
	CPG2 (17)		
	CPG3 (3)		
Diagnosis percentage core positive (%)	16.7	22.2	6.6–41
Time between first and second biopsy (days)	201	208	33–369

*Note*: Median values shown where applicable.

**TABLE 2:** Summary of reclassification and re‐grading after repeat biopsies.

Changes in disease	Change
Grade Group/CPG classified down	6 (15%)
Grade Group/CPG classified up	15 (37.5%)
Reclassified to ≥Grade Group 3	5 (12.5%
Reclassified to ≥Grade Group 4	2 (5%)
Reduction in percentage core positivity	18 (45%)
Increase in percentage core positivity	20 (50%)

Abbreviation: CPG, National Institute for Health and Care Excellence (NICE) Cambridge Prognostic Group.

These data show that despite image‐based diagnostics, a significant proportion of this cohort (37.5%) had a GG upgrade and CPG reclassification after confirmatory re‐biopsy. Most reclassifications moved men from CPG1 to CPG2 and although still eligible for AS, about 50% decide to opt for treatment instead. It is reasonable to argue that finding this out later during AS would not have impacted on eventual outcomes. However, if a centre is to employ risk‐based follow‐up and reduce over‐monitoring at the outset of AS, it has to be based on as accurate a knowledge of disease burden as possible. Here, eight of the upgraded men (24% of the whole cohort) who stayed on AS had a different and more intensive STRATCANS follow‐up after re‐biopsy. If detected later, these men would also have been wrongly labelled as disease progression instead of misclassification. Hence, getting disease assignment correct at diagnosis may reduce the apparent high progression and attrition rates noted in many AS series. Notably, of the 25 men who were not upgraded in our series, only two (8%) progressed and had treatment in subsequent AS follow‐up. There is sparse data on the role of confirmatory AS re‐biopsy after MRI diagnostics, but some corroboration can be gleaned from radical prostatectomy (RRP) comparisons. Weinstein et al.'s systematic review found a residual 27% discrepancy in histological grade from dianostic biopsy to RRP whole mount histology despite adding MRI targeted to systematic biopsies.^7^ In other reviews, GG concordance between MRI biopsies and whole mount histology was only about 60%.^8^ These figures are remarkably similar to our own finding. In the context of curative intent radical surgery or radiotherapy, this may not matter, but in AS, this is critical to get right if more aggressive disease is not to missed at the start of monitoring. In addition, getting it right the first time may abrogate the need for future protocol or triggered biopsies later in AS. This study did not reveal any specific metric that predicted reclassification but acknowledge our series is limited and underpowered to detect small differences in predictive factors; it is also potentially subject to patient choice selection bias. In summary, we propose that early repeat biopsy should be considered as an essential part of a personalised, risk stratified AS programme and should be discussed in all men embarking on AS. To not do so risks misclassification and the wrong follow‐up schedule, unnecessary imaging and biopsies, wasted appointments and potentially, a missed opportunity to treat lethal prostate cancer earlier. Further, larger studies in this area are also warranted.

## AUTHOR CONTRIBUTIONS

**Harry Gabb:** Data collection; analysis; manuscript drafting. **Vincent J. Gnanapragasam:** Conceptualization; visualisation; methodology; project administration; supervision; manuscript drafting.

## CONFLICT OF INTEREST STATEMENT

The authors declare no conflicts of interest.

## Supporting information


**Table S1** A. Analysis of diagnostic factors and potential association with likelihood of CPG re‐classification at confirmatory/early re‐biopsy. Median values shown where applicable. CPG‐ National Institute for Health and Care Excellence (NICE) Cambridge Prognostic Group. B. Analysis of initial diagnostic biopsy period and potential association with likelihood of CPG re‐classification at confirmatory/early re‐biopsy
