# Supplementary material for: Value of a confirmatory re‐biopsy as part of a modern risk stratified cancer surveillance programme for early prostate cancer
Source: BJUI Compass. 2024 Jun 1;5(7):662–4. doi: 10.1002/bco2.406 (PMC11250161; doi:10.1002/bco2.406)
Supplement: Supplementary file 1 — Table S1 A. Analysis of diagnostic factors and potential association with likelihood of CPG re‐classification at confirmatory/early re‐biopsy. Median values shown where applicable. CPG‐ National Institute for Health and Care Excellence (NICE) Cambridge Prognostic Group. B. Analysis of initial diagnostic biopsy period and potential association with likelihood of CPG re‐classification at confirmatory/early re‐biopsy [file BCO2-5-662-s001.docx]

| **Variable tested** | **Up-classified** | **Not up-classified** | p value |
| --- | --- | --- | --- |
| **PSA (ng/ml)** | 6.9 | 6.0 | 0.54 |
| **PIRADS score** | 5 | 4 | 0.67 |
| **Prostate volume (ml)** | 40 | 40 | 1.0 |
| **PSA density (ng/mL^2^)** | 0.16 | 0.12 | 0.46 |
| **Route of first biopsy** | 60% TP  40% TRUS | 32% TP  68% TRUS | 0.17 |
| **Diagnosis Percentage core positive (%)** | 16.7% | 16.7% | 1.0 |
| **Time between 1^st^ and 2^nd^ biopsy (days)** | 195 | 206 | 1.0 |

**A**

| **Changes in disease** | **2018-2021** | **2021-2022** | **p value** |
| --- | --- | --- | --- |
| **Percentage CPG classified up** | 35% | 46% | 0.74 |
| **Percentage Increase in core positivity** | 60% | 46% | 0.12 |

**B**

**Supplementary Table S1 A.** Analysis of diagnostic factors and potential association with likelihood of CPG re-classification at confirmatory/early re-biopsy. Median values shown where applicable. CPG- National Institute for Health and Care Excellence (NICE) Cambridge Prognostic Group. **B.** Analysis of initial diagnostic biopsy period and potential association with likelihood of CPG re-classification at confirmatory/early re-biopsy
